# Digital Interventions for Suicide Prevention: Systematic Review and Meta-Analysis of RCTs Examining Age, Gender, and Control Group Type

**DOI:** 10.1027/0227-5910/a000996

**Published:** 2025-04-04

**Authors:** Sean K. Burr, Miao Yu, Danny Clark, Dana Alonzo, Robin E. Gearing

**Affiliations:** ^1^ Graduate College of Social Work, University of Houston, Houston, TX, USA; ^2^ Division of Public Health Pediatrics, Baylor College of Medicine, Houston, TX, USA; ^3^ Diana R. Garland School of Social Work, Baylor University, Waco, TX, USA; ^4^ Graduate School of Social Service, Fordham University, West Harrison, NY, USA

**Keywords:** suicide prevention, suicidal ideation, digital intervention, systematic review, meta-analysis

## Abstract

**Abstract:** *Background:* Digital-based mobile interventions hold significant promise in preventing suicide. Although mixed, some evidence suggests these interventions are effective and capable of overcoming barriers such as cost and stigma. *Aim(s):* This review aimed to determine the effectiveness of digital interventions designed to address suicidal ideation and behaviors and the impacts of age, gender, and control group type on these outcomes. *Methods:* Databases were searched for randomized controlled trials (RCTs) on digital suicide interventions (apps/online programs) published before January 1, 2022. Data were analyzed using a random-effects model in Stata 17. *Results:* The search identified 4,317 articles, and 16 were included. Risk of bias analysis found studies to be of low-to-moderate quality. The random-effects model indicated a small but significant effect of treatment on suicidal ideation, *k* = 16, *g* = 0.11 (95% CI: 0–0.23), *p* = .049. Subgroup analyses found the interventions to have a significant effect on adults (*g* = 0.15, 95% CI: 0.03, 0.28, *p* = .01) but not adolescents. The interventions showed better effects compared to waitlist controls (*g* = 0.28, 95% CI: 0.19, 0.38) but not compared to treatment as usual or active controls [χ^2^(2) = 29.41, *p* < .001]. *Limitations:* Sample sizes across studies were insufficient for examining the effectiveness of digital interventions by gender. Limited studies reported on suicidal behaviors, so the impact of digital interventions on these behaviors could not be analyzed. *Conclusions:* This review found a significant effect of digital interventions for reducing suicidal ideation and highlights the importance of examining the effectiveness across subgroups.

Suicides and suicidal behavior are complex and multifaceted public health phenomena that incur tremendous social and economic cost (De Berardis et al., 2018). The World Health Organization (WHO) estimates that, globally, more than 700,000 individuals die by suicide annually, with many more making suicide attempts (WHO, 2021). Low- and middle-income countries (LMICs) account for 77% of global suicides, yet high-income countries have the highest suicide rate (WHO, 2021). Suicide rates vary significantly across countries and also differ significantly by sex, age, and means (WHO, 2021). The ubiquity and ongoing persistence of suicide along with the variation of suicidal behaviors require suicide prevention and intervention approaches to adapt and evolve (Zalsman et al., 2016). One area of innovation has been digital suicide prevention interventions that are self-guided mobile phone applications (apps) and online programs, which have increasingly been developed in recent years (Connolly et al., 2021). Furthermore, the COVID-19 pandemic has reinforced the utility of digital tools in healthcare systems (Connolly et al., 2021). Digital interventions for suicide prevention hold the potential to overcome certain access barriers for traditional treatment and act as complementary or standalone interventions for addressing suicidal behaviors.

Despite a diversity in digital interventions for suicide prevention, most share common functions and features and are designed to be used independently of professional support (Torok et al., 2020). A review of mobile technologies for suicide prevention identified the following key features across interventions: education, resource locators (i.e., local medical facilities), emergency buttons (direct connection to a crisis line), safety planning, coping tools, clinical assessment (via self-report or ecological momentary assessment), and automated interventions relating to assessed risk (Luxton et al., 2015). Nascent research indicates positive attitudes toward mental health apps and online interventions. Reyes-Portillo et al. (2022) surveyed college students about their attitudes toward mental health apps and online interventions and found that most students (65%) were motivated to use them. Additionally, they found students with suicidal ideation were most inclined to use digital interventions compared to students with other mental health issues (Reyes-Portillo et al., 2022).

Research indicates that individuals who die by suicide exhibit low levels of help-seeking. A review of 35 empirical studies reporting prevalence of contact with mental health services before suicide found only 25.7% (CI = 22.7%–28.9%) of individuals had contact with outpatient or inpatient services in the year prior (Walby et al., 2018). Similarly, Hom et. al (2015) found that only 29.5% of individuals with suicidal behaviors (ideation, plans, or attempts) in the past year sought or engaged mental health services. Possible drivers for these lower rates of help-seeking include fear of hospitalization, stigma, and preference for self-management (Hom et al., 2015; WHO, 2021). Digital interventions may be uniquely positioned to address many of these barriers given their offer of anonymity, choice, and self-pacing.

Globally, 75% of individuals needing mental health services lack access often due to cost, availability of qualified practitioners, and geography (Patel et al., 2011). The mental health treatment gap is particularly pronounced in LMICs (Patel et al., 2011). Digital interventions, provided they are priced affordably, could address these structural issues of cost and availability. It is estimated that over half of the global population have access to smartphones (O’Dea, 2021). Mobile data subscribership is estimated to have penetrated 73% of the global market, and rates across low-, middle-, and high-income countries continue to rise (O’Dea, 2021). The widespread and growing use of smartphones across countries and social segments indicates that digital interventions for suicide prevention could reach groups such as those living in remote or rural areas more easily than traditional mental health services provided there is reliable connectivity to high-speed internet and mobile services.

Despite the apparent feasibility of smartphones for the delivery of suicide prevention interventions, the landscape for mental health apps is complex for the individual consumer. It has been estimated that there are over 10,000 mental health- and psychiatry-related apps available (Connolly et al., 2021). Whereas limited regulations exist for digital interventions themselves, across countries various regulations constrict their use in standard healthcare practice (Connolly et al., 2021). Since the pandemic, some countries (e.g., India, Germany, the United States) have created new regulations that support greater use of digital tools in healthcare settings. An updated understanding of the effectiveness of digital interventions for suicide prevention is critical, not only for the individual consumer but also for policy-makers and clinicians who are determining how they could be incorporated into existing models of care.

## Existing Systematic Reviews of Web and Mobile Applications for Suicide Prevention

Recent meta-analyses/systematic reviews of digital interventions have found varied results on the clinical effectiveness of these interventions. Torok et al.’s (2020) systematic review and meta-analysis investigated whether digital interventions targeting suicidal behaviors and interventions targeting depression were effective in reducing suicidal ideation. All interventions were found to be effective in reducing suicidal ideation but interventions that targeted suicidal ideation were found to be more effective compared to those targeting depression (Torok et al., 2020). This review confirmed the effectiveness of digital interventions, specifically those with direct suicide prevention content. However, Arshad et al.’s (2020) meta-analysis examined 11 randomized controlled trials (RCTs) of digital interventions for self-injurious thoughts and behaviors and found nonsignificant treatment effects for suicidal ideation and suicide attempts. Similarly, inconsistent results were found in a review of 14 RCTs examining the effectiveness of digital interventions in reducing self-harm and suicidal ideation leading the authors to conclude there is insufficient research to warrant their consideration as evidence-based treatments (Stefanopoulou et al., 2020). A more recent meta-analysis by Sutori et al. (2024) found insufficient evidence for digital interventions impacting death by suicide and nonsignificant findings for their impact on suicide attempts. For the outcome of suicidal ideation, the authors found a small but favorable effect. A 2024 meta-analysis by Oh and colleagues found a significant and moderate effect of digital interventions on suicidal ideation and on other suicidal variables (i.e., suicide risk and severity as outcomes).

Despite some consensus in the research on the positive effects of digital interventions on suicidal ideation, the current evidence is limited in quantity and has yet to clearly establish the effectiveness of these interventions. Moreover, a recent systematic review of suicide prevention apps identified primary concerns relating to the design (e.g., lack of evidence-based sources), efficacy (e.g., low user engagement, high dropout rates of participants), and evaluation (e.g., small sample sizes, short evaluation periods) of these interventions (Jha et al., 2024). Given the potential of digital interventions for suicide prevention (e.g., overcoming barriers relating to access and stigma), it is critical that they be rigorously appraised for their effectiveness. To address existing inconsistencies, the current review examines the effectiveness of digital interventions for suicide prevention designed to target suicidal ideation and behaviors. Additionally, existing reviews have paid minimal attention to understanding the effect of these interventions on different subgroups. As research has clearly established differential rates of suicide and suicidal behavior, and the effectiveness of interventions across age groups and genders, the current review examines the influence of common sociodemographic and treatment factors on the effectiveness of the digital interventions, specifically sample age (adolescents vs. adults), gender (male vs. female), and control group type (treatment as usual [TAU] vs. waitlist vs. active control).

## Methods

### Search Strategy

A search for English-language peer reviewed journal articles was conducted across five databases. The searches were restricted to articles published anytime up until January 1, 2022. The search (see Electronic Supplementary Material 1 [ESM 1] for full details) comprised three search strings and included key words relating to digital and mobile technologies, suicide and suicidal behaviors, and interventions and treatment. The keywords for strings one and two were limited to abstracts, and the third string was left open (title, abstract, body). The PRISMA reporting guidelines were adhered to, and the protocol for the review was preregistered with PROSPERO (CRD42021230901).

## Inclusion and Exclusion Criteria

Published RCTs of the effectiveness of suicide prevention interventions in the form of mobile apps or internet-based programs that can be utilized with mobile/cell phone technology were included. The intervention needed to have been designed or adapted to specifically address suicidal ideation or suicidal behaviors (planning or attempts) or both. A primary outcome of a suicide variable needed to be reported. The search criteria excluded the following: (1) review, meta-analyses, and systemic review articles; (2) letters, opinions, and commentaries on the use of technology in suicide prevention; (3) articles on the design or usability of apps or online suicide prevention interventions; (4) nonintervention studies; and (5) studies that investigated digital interventions for other mental health concerns (i.e., depression).

## Potential for Bias

Risk of bias was assessed for the included studies and conducted by two members of the research team. Risk of bias within selected studies was assessed using the Cochrane risk of bias tool for RCTs (Higgins et al., 2011). Assessments were completed independently, and final assignments of risk of bias were determined by consensus.

## Data Extraction

Identified abstracts were reviewed by a team of authors and divided into three categories: ineligible, eligible, and uncertain. Articles categorized as uncertain were returned to the team for discussion and consensus. Full-text articles were then reviewed, and data abstraction forms were completed. The authors extracted data using a custom spreadsheet to record the study design, characteristics of the intervention (therapeutic model, duration, intensity), delivery approach (mobile app, text, website), study sample (sample size, characteristics, sex), age (adolescents, adults), comparison condition (TAU, active control, waitlist), study outcomes (suicidal ideation, suicide plans, suicide attempts), and missing data handling (intent-to-treat vs. completer data). Note, the second type of control group was active control, defined as other treatment types that are more than usual care. For age, the delineation between adults and adolescents was determined by the lower and upper age limits as defined across studies as well as by the intended age group targeted by the intervention (if applicable).

## Meta-Analysis

Extracted data were analyzed using a random-effects Restricted Maximum Likelihood (REML) model in Stata 17. The random-effects model was adopted as heterogeneity in the treatment model as expected. After reviewing the final articles, it was determined that suicidal ideation would be the primary and sole outcome variable for this review. Suicidal ideation was reported on in all included studies, whereas suicidal behaviors were only reported on in a third of studies, which was not enough to undertake a meta-analysis. The analysis focused on the most immediate time point post treatment. In most studies, suicidal ideation was measured as a continuous construct with different levels of severity or frequency. Raw (unstandardized) change scores of suicidal ideations from preintervention to post-treatment were first calculated for each intervention condition (intervention vs. control). The standard deviation (*SD*) of a change score was calculated from the *SD* of preintervention and *SD* of postintervention with the correlation between preintervention score and postintervention score (*r*) set as 0.5. A standardized change score from preintervention to postintervention for each treatment condition was then calculated from the raw change score and the *SD* of change score. For studies that reported binary results for suicidal ideation, the odds ratio was converted to a standardized mean difference (*d*). Based on the standardized change score for each treatment condition, Hedges’ *g* was calculated to represent the effect size of the intervention group compared to the control group. A forest plot was presented along with the heterogeneity statistics.

Subgroup analyses were planned a priori and included comparisons of the age category of study participants (adolescents vs. adults), missing data handling (ITT vs. non-ITT), and control group type (waitlist vs. TAU vs. active control). A meta-regression analysis was performed to examine the relation between the intervention effect and the percentage of male participants. Two sensitivity analyses were conducted. One sensitivity analysis changed the correlation between preintervention and postintervention (*r*) from 0.5 to 0.7. The second sensitivity analysis was conducted by comparing the post-intervention scores (endpoint data) between the intervention group and the control group.

## Results

### Search Results

**Figure 1:**
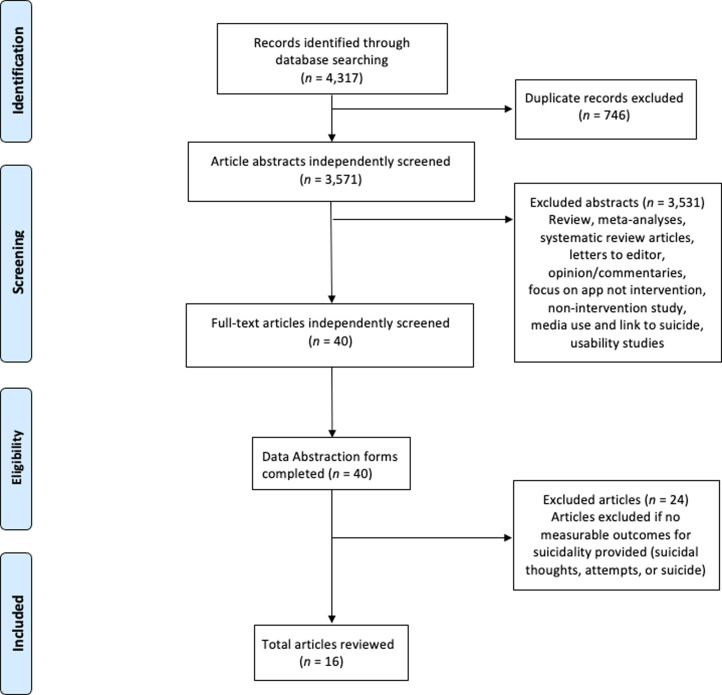
PRISMA flow diagram.

A total of 4,317 articles were identified and imported into EndNote 20 (EndNote Team, 2013). After the removal of duplicate records, the abstracts of 3,571 articles were independently screened by three authors (DA, RG, SB), which resulted in 3,531 articles being removed according to the exclusion criteria. The remaining 40 articles were independently screened, and data abstraction forms were completed. An additional 24 articles were identified and excluded due to an absence of a measurable suicidal outcome or lacking a randomized controlled design. Data were extracted from the final 16 articles by two team members into a custom spreadsheet (see Table 1). Data were missing from two articles, and the research team contacted the authors who provided the requested information. Figure 1 provides the PRISMA flow diagram.

**Table 1:** Study characteristics of included studies^a^

Author(s), country	Study design	Baseline sample size	Sample characteristics	Age in years *M* (*SD*)	Male	Intervention	Control condition	Delivery approach	ITT	Primary outcome measures
Battterham et al. (2018), Australia	RCT	128	Adults with symptoms of depression, anxiety, suicidal ideation, or substance use, but no prior suicide attempt	Range: 18–65+	19% for intervention, 11% for control	FitMindKit	Attention control: Healthwatch program	Online	Yes	Suicidal Ideation Attributes Scale (SIDAS)
Battterham et al. (2021), Australia	RCT	1986	Adults with psychological distress and/or risk of a mental disorder	Range: 18–65+	15% for intervention, 15% for control	FitMindKit	Attention control: Healthwatch program	Online	Yes	SIDAS
Bush et al. (2017), USA	RCT	118	Adult veterans with suicidal ideation; majority reported depression or PTSD	46.5 (13.8) intervention, 48.7 (14.3) control	62% for intervention, 75% for control	Virtual Hope Box + TAU	TAU	Mobile app	Yes	Beck Scale for Suicidal Ideation (BSS)
de Jaegere et al. (2019), Belgium	RCT	724	Adults with suicidal ideation	35.7 (13.6)	59.40%	Think life	Waitlist with online information resource	Online	Yes	BSS
Dobias et al. (2021), USA	RCT	565	Youth	14.95 (0.98)	8.70%	Stop Adolescent Violence Everywhere (SAVE)	Supportive therapy	Online	Yes	Frequency count of suicidal ideation
Franklin et al. (2016), Study 3, USA^b^	RCT	163	Adults with suicidal behavior in last year	24.5 (6.6)	41%	Therapeutic Evaluative Conditioning (TEC)	Nonactive version of TEC	Mobile app	No	Self-Injurious Thoughts and Behaviors Interview (SITBI)
Hetrick et al. (2017), Australia	RCT	50	High school students with recent suicidal ideation	14.7 (1.4)	82%	Reframe-IT + TAU	TAU	Online	No	Suicidal Ideation Questionnaire (SIQ)
Kennard et al. (2018), USA	RCT	66	Adolescents recently hospitalized for suicidal ideation or attempt	15.1 (1.5)	11%	ASAP + TAU	TAU	Mobile app	Yes	Suicidal Ideation (SIQ), Suicide attempt (Yes/No)
Li et al., (2019), China	RCT	300	Outpatient adults with depressive symptom	27.5 (1.18)	92.30%	Run4Love	Waitlist	Mobile app	No	Suicidal ideation (Yes/No), attempt (Yes/No)
Muhlmann et al. (2021), Denmark	RCT	402	Adults with suicidal ideation	32.8 (SD = 13) intervention, 34.3 (13) control	71%	Living under control	Waitlist	Online	Yes	BSS
O'Toole et al. (2019), Denmark	RCT	129	Outpatients with suicidal ideation	28.1 (9.2) intervention, 29.3 (9.7) control	60% for intervention, 56% for control	APP + TAU	TAU	Mobile app	Yes	Suicide Status Form II–R (SSF)
Rodante et al. (2020), Argentina	Cluster RCT	21	Adults with suicidal ideation, suicidal plan, attempt, gesture, or self-injurious behavior	31.8 (6.94) intervention, 27.7 (6.6) control	64% for intervention, 100% for control	CALMA + DBT	DBT	Mobile app	No	SITBI
Tighe et al. (2017), Australia	RCT	61	Youth with depression and suicidal ideation	27.5 (9.54) intervention, 25 (6.28) control	35% for intervention, 37% for control	iBobbly	Waitlist	Mobile app	Yes	Depressive Symptom Inventory-Suicidality Subscale (DSI-SS)
Van Spijker et al. (2014), Netherlands	RCT	236	Adults with suicidal ideation	40.9 (13.71)	66.10%	Web-based self-help	Information waitlist	Online	Yes	BSS
Van Spijker et al. (2018), Australia	RCT	418	Adults with suicidal ideation	40.6 (11.9)	77.30%	Web-Based Self-Help	Information waitlist	Online	Yes	Columbia Suicide Severity Rating Scale (C-SSRS)
Wilks et al. (2018), USA	RCT	59	Individuals with heavy episodic drinking	38.0 (10.4)	69.50%	i-DBT	Waitlist	Online	Yes	Scale for Suicidal Ideation (SSI)
*Note*. ^a^Full citations provided in Electronic Supplementary Material 1 (ESM 1). ^b^Intervention modified to target suicidal behaviors.										

### Potential for Bias

Utilizing the Cochrane risk of bias tool, the evaluators found that most studies had a lower risk of bias overall with attrition (Domain 3) being the highest concern (see Table E1 in ESM 1). Within the results of the 16 studies, six studies (35.7%) were found to have some risk associated with attrition. Four studies had more than 20% attrition after initial treatment or follow-up. Five studies provided details related to the handling of missing data with one not clearly indicating potential bias attributable to incomplete data.

### Study Characteristics

The 16 studies provided unique comparisons utilizing baseline data on 5,426 participants. Sample characteristics varied across clinical and nonclinical groups and across community, inpatient, and outpatient settings (Table 1). Sample sizes ranged from 21 to 1,986 participants (*M* = 339.13, *SD* = 484.26), and nine studies (56%) had sample sizes of fewer than 200 participants. Roughly a third of the studies were from Australia and the United States, and the remainder were from other countries: Argentina, Belgium, China, Denmark, and the Netherlands. Most of the adult studies had predominately male samples, and most of the adolescent studies had predominately female samples All included studies targeted either adolescents or adults. These categories were mutually exclusive, with *adolescents* defined as persons between the ages of 12–17 years, and *adults* defined as persons 18 years and older. One adolescent study with youth from 13 years through to the end of 18 years (*M*_age_ of 14.7 years) was included within the adolescents’ group as the intervention was designed for this age group (Hetrick et al., 2017). The range of mean ages in adult studies was 24.5 years to 46.5 years (*SD* = 6.9) and 14.7 years to 15.1 years in adolescent studies. Intention-to-treat analysis was present in most studies (75%). With respect to the delivery approach, more interventions were provided online (56%) than by mobile app (44%).

### Findings

**Figure 2:**
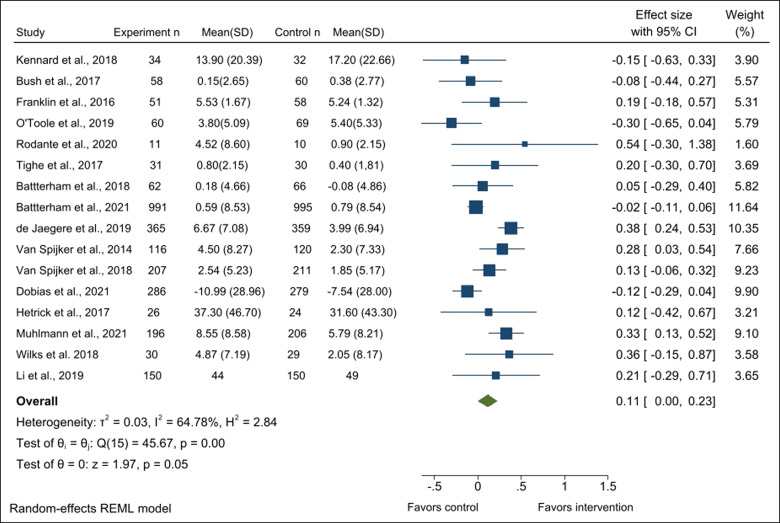
Forest plot of treatment effect on suicidal ideation.

A random-effects model indicated a small but significant effect of treatment on suicidal ideation, *k* = 16, *g* = 0.11 (95% CI: 0.0–0.23), *p* = .049. The test of heterogeneity showed that the effect sizes among studies varied significantly, as expected (Figure 2). No studies were found to report negative results from digital interventions.

**Figure 3:**
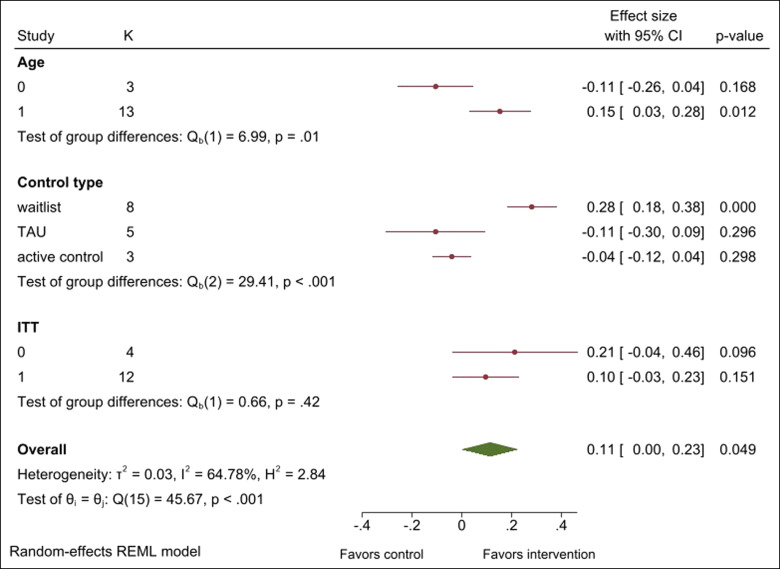
Subgroup analyses with age, control type, and ITT.

A set of subgroup analyses were conducted to investigate the influence of age, missing data handling, and control type and gender on the effectiveness of digital interventions for suicidal ideation. Figure 3 shows the digital interventions had a significant effect on adults (*g* = 0.15, 95% CI: 0.03, 0.28, *p* = .01) while no significant effect on adolescents (*g* = −0.11, 95% CI: −0.26, 0.04, *p* = .17). The difference of effect between adults and adolescents was significant [*Q*_b_(1) = 6.99, *p* = .01]. With respect to control group type, there was a significant difference between the three groups [χ^2^(2) = 29.41, *p* < .001]. The digital interventions showed better effects compared to waitlist controls (*g* = 0.28, 95% CI: 0.19, 0.38) but no significant difference compared TAU (*g* = −0.11, 95% CI: −0.31, 0.10) or to active control (*g* = −0.04, 95% CI: −0.12, 0.04). The effectiveness of digital interventions for suicide ideation did not differ significantly based on how missing observations were handled. Meta-regression analysis was conducted to investigate the relation between gender of the participants and the effect of digital interventions. The proportion of male participants in each study was used as a continuous predictor of the effect size. No significant relation (*b* = 0.002, standard error = 0.003, *p* = .64) was observed between the proportion of male participants and the effect size across studies.

## Discussion

This review examined the effectiveness of digital interventions targeting suicidal ideation based on 16 RCTs with 5,426 participants. To date, this is the most comprehensive meta-analysis of digital interventions designed for suicide prevention in terms of number of RCTs and participants. Also, it is the first identified meta-analysis to explore the effectiveness of these interventions across the specific sociodemographic and treatment factors of gender, age, and type of control group. Despite the low-to-moderate quality of the studies included, a small but significant effect of treatment on suicidal ideation was observed (*g* = 0.11, 95% CI: 0.0, 0.23, *p* = .049). Subgroup analyses for age found digital interventions to be effective for adults but not for adolescents in reducing suicidal ideation. Furthermore, digital interventions were found to be more effective than waitlist controls but showed no significant difference compared to TAU or active controls. For gender, no significant relationship was observed due to the limitations in power across identified studies.

With a larger sample size (e.g., studies, participants) and more nuanced focus on treatment factors (e.g., age, type of control group), these findings uniquely contribute to an understanding of digital interventions and their possible use in suicide prevention. This review’s main finding identified a significant albeit small postintervention effect for suicidal ideation. This echoes the results found by two other recent reviews (Sutori et al., 2024; Torok et al., 2020), and contrasts slightly with Oh et al. (2024) who also found significant results but with moderate effects, which may be in part due to a lower number of studies (*k* = 9). However, this finding differs with Arshad et al. (2020) who did not find a positive treatment effect on suicidal ideation, due perhaps to differing aims as their meta-analysis included interventions for nonsuicidal self-injury.

Comparing and situating digital interventions and their efficacy within the existing landscape of suicide prevention strategies is complex given limited RCTs in this area and that existing evidence indicates no single approach is clearly superior (Zalsman et al., 2016). A possible comparator for digital interventions could be brief face-to-face interventions for suicide prevention that contain similar components to digital interventions (i.e., information provision, safety planning). A review by McCabe et al. (2018) on brief psychological interventions for suicidal presentations did not identify any trials that had an impact on suicidal ideation. A more recent meta-analysis of community and clinical interventions targeting suicide attempts, self-harm, and suicidal ideation found a significant but small effect size on measures of suicidal ideation within identified trials (*g* = 0.47; Gaynor et al., 2023). The implications of this review’s findings suggest that digital interventions are at a sufficient level of evidence within the existing field of suicide prevention to warrant their consideration for broader application. Considering lifetime global prevalence rates for suicidal ideation is roughly 9.2% (Nock et al., 2008), digital interventions could have a significant population-level impact. This stated the interventions in this review were developed and tested in different settings and countries, so larger implementation research is needed for each context intended for dissemination.

Results from the subgroup analyses of control group type further suggest digital interventions could be more seriously considered for broader use. Digital interventions were found to be significantly more effective than waitlist controls but not significantly more effective than TAU or active controls. These results mirror those found by other authors (Torok et al., 2020; Witt et al., 2017) who also found a greater magnitude of intervention effects associated with waitlist controls compared to active/attention controls. An interpretation of these similar findings across meta-analyses is that digital interventions are more effective than null, which equates to the minimum standard of care ethically permitted in trials. Considering that many countries’ median wait times for psychiatric assessment and treatment are measured in months, and prolonged wait times have been associated with treatment outcomes that are less favorable, these findings suggest digital interventions could provide initial treatment for individuals experiencing suicidal ideation as they wait for more formal mental health care (van Dijk et al., 2023). Also, research indicates that some individuals with suicidal ideation prefer online methods of support compared to face-to-face methods, and so digital interventions could facilitate initial engagement and possibly act as a bridge to more formal in-person treatment (Wilks et al., 2018).

Digital interventions could also be well suited to non-treatment-engaged individuals with suicidal ideation, a population that may be neglected due to barriers such as stigma or fears of hospitalization (Ward-Ciesielski et al., 2017). Digital interventions could provide this group the opportunity for some degree of treatment that is anonymous and self-managed even if they never engage in formal care (Ward-Ciesielski et al., 2017). These interventions could be a complement to existing suicide prevention strategies as multilevel/multiple interventions have been similarly shown to have a synergistic and additive effect towards reducing suicide (Hofstra et al., 2020). Further research should investigate the effects of digital interventions when added to existing suicide prevention plans that include multiple approaches (i.e., gatekeeper training, media campaigns, etc.) as they may strengthen the overall effects of these other efforts (Hofstra et al., 2020).

The subgroup finding relating to adolescents not seeing a treatment effect was unanticipated given the higher ownership and usage within this age group (Jensen et al., 2019). Concepts such as *technostress* are used to describe stress associated with using information and communications technology that have been found to contribute to learning burnout for young people in technology-enhanced learning settings (Upadhyaya & Vrinda, 2021). This phenomenon relating to the overuse of technology may impact the effectiveness of these interventions, although research finds teenagers engage in web-based conversations about suicide more often than adults, so perhaps the barrier relates to how the intervention is presented digitally (Sweeney et al., 2024). Given that Torok et al. (2020) similarly found no evidence for an effect of digital interventions for adolescents, it is recommended that more nuanced research examine specific mechanisms of apps or online programs for this group.

This review’s finding of a significant effect of digital interventions for reducing suicidal ideation in adults supports previous findings (Torok et al., 2020) and provides additional support for targeting adults who account for 52% of suicides globally (Qin et al., 2022). Suicides during adulthood are strongly associated with socioeconomic difficulties as well as physical and psychiatric illnesses (Qin et al., 2022). This life period can be characterized by high social, economic, and familial expectations and so barriers such as cost and time could be mitigated provided mobile apps/online interventions are affordable (Hom et al., 2015). Targeting dissemination of digital interventions for adult populations in LMICs could be particularly effective given the treatment gap for psychological/psychiatric treatments in many of these countries (Patel et al., 2011). This review only identified two RCTs from developing countries (Argentina and China) and so further research is required to determine the effectiveness and delivery of digital interventions in low resource settings.

There was insufficient power to examine the effectiveness of digital interventions by gender. There is a widely accepted relationship between gender and suicide risk, with males dying by suicide more often and females attempting suicide more often (WHO, 2021). This relationship is nuanced and complex, and when considering the dissemination of digital interventions, further research should investigate how gender may impact the usability and overall intervention effectiveness.

### Limitations

There were limitations related to the methodological nature of existing research. Studies largely had small samples resulting in low power, limiting our ability to analyze a large range of potential mediators/moderators. Given that research has established that individuals who think about suicide have different characteristics than those engaging in suicidal behavior, our findings highlight the need for people who have attempted suicide to be included in future RCTs. Also, the samples were largely non-diverse, limiting our ability to analyze effectiveness across race/ethnicity. Although publication bias did not emerge as an issue, the effectiveness of mobile apps/online programs may be overestimated if nonsignificant suicide ideation outcomes were not reported. There was also heterogeneity in the outcome measures used across studies. This limits our ability to confidently determine that the varied studies were indeed measuring the same constructs. Lastly, as this is an emerging area in suicide prevention, it is important to recognize that interventions continue to be developed (i.e., Rainbow et al., 2024). The search cut-off date for the current review was January 1, 2022, so ongoing evaluative research is necessary to stay up-to-date on the effectiveness of these interventions.

### Conclusion

The current review establishes digital interventions as a viable approach for reducing suicidal ideation. Digital interventions are effective for adult populations and have the potential to benefit people with no other treatment or support options. These findings warrant further research into their acceptability and safety for individual consumers and use within healthcare settings. Such research should be prioritized as these interventions hold the potential of making a significant impact in low-resource contexts. Additionally, as the evidence base for digital interventions is still developing, further testing and refinements are necessary to ensure the progress of this emerging area in the field of suicide prevention.

## Electronic Supplementary Material

The electronic supplementary material is available with the online version of the article at https://doi.org/10.1027/0227-5910/a000996

**ESM 1.** Figure shows the search algorithm used for the systematic search. Table shows the results of the Cochrane risk-of bias tool for randomized trails. Bibliographic information for included studies.

## Notes

**Authorship:** Sean K. Burr, writing – original draft writing, visualization, conceptualization, project administration; Miao Yu, methodology, formal analysis, data curation, visualization, writing – review & editing; Danny Clark, formal analysis, visualization, writing – original draft; Dana Alonzo, conceptualization, methodology, writing – original draft, writing – review & editing; Robin E. Gearing, conceptualization, methodology, project administration, writing – review & editing. All authors approved the final version of the article.

**Open Science:** Preregistration and Analysis Plan: This study was preregistered at PROSPERO: CRD42021230901.
