# Supplementary material for: Digital Interventions for Suicide Prevention: Systematic Review and Meta-Analysis of RCTs Examining Age, Gender, and Control Group Type
Source: Crisis. 2025 Apr 4;46(3):176–86. doi: 10.1027/0227-5910/a000996 (PMC12096958; doi:10.1027/0227-5910/a000996)
Supplement: Supplementary file 1 [file cri_46_3_176_esm1.pdf]

https://doi.org/10.1027/0227-5910/a000996

Electronic Supplementary Material 1 for "Digital Interventions for Suicide Prevention" by Sean K. Burr et al.

Figure E1. Search algorithm used for the systematic search.

Databases: PsycINFO, Medline, PubMed, CINHAL, The Cochrane Library
Keywords (strings 1-2, terms limited to abstract; string 3, open to anywhere in article (title, abstract, body)):

Search Sting 1: web or online or mobile device or tablet computer or iPad or iPhone or Samsung or android or windows or phone app or mobile application or smartphone application or mobile phone or cell phone or smartphone or digital or eHealth or mHealth or mental health technology or mobile technology or mobile apps or telehealth or telemedicine

Search String 2: suicid*

Search String 3: intervention or strategies or best practice or treatment or therapy or therapeutic or program or management or care

Limits:
- Years: Open
- Language: English
- Source: Peer reviewed journal articles

© 2025 The Author(s). Distributed as a Hogrefe OpenMind article under the license CC BY 4.0 (https://creativecommons.org/licenses/by/4.0)

https://doi.org/10.1027/0227-5910/a000996

Table E1. Results of the Cochrane risk-of bias tool for randomized trails (Rob2).

Study	Randomisation	Deviations from intended intervention	Missing outcome data	Measurement of the outcome	Selection of the reported result	Overall
Bush, 2017	Low	Low	Low	Low	Low	Low
Franklin, 2016 (Study 3)	Low	Low	Low	Low	Low	Low
Kennard, 2018	Low	Some Concern	Low	Low	Low	Some Concern
Li, 2019	Low	Low	Low	Low	Low	Low
O'Toole, 2019	Low	Low	Low	Some Concern	Low	Low
Rodante, 2020	Some Concern	Low	Low	Some Concern	Low	Some Concern
Tighe, 2017	Low	Low	Low	Low	Low	Low
Battterham et al., 2018	Low	Low	Low	Low	Low	Low
Battterham et al., 2021	Low	Low	Low	Low	Low	Low
de Jaegere et al., 2019	Low	Low	Low	Low	Low	Low
Dobias, 2021	Low	Low	Low	Low	Low	Low
Hetrick et al., 2017	Low	Some Concern	Low	Low	Low	Some Concern
Muhlmann, 2021	Low	Low	Low	Low	Low	Low
Van Spijker et al., 2014	Low	Low	Low	Low	Low	Low
Van Spijker et al., 2018	Low	Low	Low	Low	Low	Low
Wilks et al. 2018	Low	Low	Low	Low	Low	Low
% with Concern	6.25%	12.50%	0%	12.5%	0%	18.8%

© 2025 The Author(s). Distributed as a Hogrefe OpenMind article under the license CC BY 4.0 (https://creativecommons.org/licenses/by/4.0)

https://doi.org/10.1027/0227-5910/a000996

Full bibliographic information for studies included in the analyses.

Bush, N. E., Smolenski, D. J., Denneson, L. M., Williams, H. B., Thomas, E. K., & Dobscha, S. K. (2017). A virtual hope box: randomized controlled trial of a smartphone app for emotional regulation and coping with distress. *Psychiatric Services*, *68*(4), 330-336. https://doi.org/10.1176/appi.ps.201600283

Franklin, J. C., Fox, K. R., Franklin, C. R., Kleiman, E. M., Ribeiro, J. D., Jaroszewski, A. C., ... & Nock, M. K. (2016). A brief mobile app reduces nonsuicidal and suicidal self-injury: Evidence from three randomized controlled trials. *Journal of Consulting and Clinical Psychology*, *84*(6), 544-557. https://doi.org/10.1037/ccp0000093

Kennard, B. D., Goldstein, T., Foxwell, A. A., McMakin, D. L., Wolfe, K., Biernesser, C., ... & Brent, D. (2018). As Safe as Possible (ASAP): a brief app-supported inpatient intervention to prevent postdischarge suicidal behavior in hospitalized, suicidal adolescents. *American journal of psychiatry*, 175(9), 864-872. https://doi.org/10.1176/appi.ajp.2018.17101151

Li, Y., Guo, Y., Hong, Y. A., Zhu, M., Zeng, C., Qiao, J., ... & Liu, C. (2019). Mechanisms and effects of a WeChat-based intervention on suicide among people living with HIV and depression: Path model analysis of a randomized controlled trial. *Journal of medical internet research*, *21*(11), e14729. doi:10.2196/14729

O'Toole, M. S., Arendt, M. B., & Pedersen, C. M. (2019). Testing an app-assisted treatment for suicide prevention in a randomized controlled trial: effects on suicide risk and depression. *Behavior therapy*, *50*(2), 421-429. https://doi.org/10.1016/j.beth.2018.07.007

Rodante, D. E., Kaplan, M. I., Olivera Fedi, R., Gagliesi, P., Pascali, A., José Quintero, P. S., ... & Daray, F. M. (2022). CALMA, a mobile health application, as an accessory to therapy for reduction of suicidal and non-suicidal self-injured behaviors: a pilot cluster randomized controlled trial. *Archives of suicide research*, *26*(2), 801-818. https://doi.org/10.1080/13811118.2020.1834476

Tighe, J., Shand, F., Ridani, R., Mackinnon, A., De La Mata, N., & Christensen, H. (2017). Ibobbly mobile health intervention for suicide prevention in Australian Indigenous youth: a pilot randomised controlled trial. *BMJ open*, *7*(1), e013518. http://dx.doi.org/10.1136/bmjopen-2016-013518

Batterham, P. J., Calear, A. L., Farrer, L., McCallum, S. M., & Cheng, V. W. S. (2018). FitMindKit: Randomised controlled trial of an automatically tailored online program for mood, anxiety, substance use and suicidality. *Internet interventions*, 12, 91-99. https://doi.org/10.1016/j.invent.2017.08.002

Batterham, P. J., Calear, A. L., Farrer, L., Gulliver, A., & Kurz, E. (2021). Efficacy of a transdiagnostic self-help internet intervention for reducing depression, anxiety, and suicidal ideation in adults: Randomized controlled trial. *Journal of medical Internet research*, *23*(1), e22698. doi:10.2196/22698

De Jaegere, E., van Landschoot, R., Van Heeringen, K., van Spijker, B. A., Kerkhof, A. J., Mokkenstorm, J. K., & Portzky, G. (2019). The online treatment of suicidal ideation: A randomised controlled trial of an unguided web-based intervention. *Behaviour Research and Therapy*, *119*, 103406. https://doi.org/10.1016/j.brat.2019.05.003

Dobias, M. L., Schleider, J. L., Jans, L., & Fox, K. R. (2021). An online, single-session intervention for adolescent self-injurious thoughts and behaviors: Results from a randomized trial. *Behaviour research and therapy*, *147*, 103983. https://doi.org/10.1016/j.brat.2021.103983

© 2025 The Author(s). Distributed as a Hogrefe OpenMind article under the license CC BY 4.0 (https://creativecommons.org/licenses/by/4.0)

https://doi.org/10.1027/0227-5910/a000996

Hetrick, S. E., Yuen, H. P., Bailey, E., Cox, G. R., Templer, K., Rice, S. M., ... & Robinson, J. (2017). Internet-based cognitive behavioural therapy for young people with suicide-related behaviour (Reframe-IT): a randomised controlled trial. *BMJ Ment Health*, *20*(3), 76-82. http://dx.doi.org/10.1136/eb-2017-102719

Mühlmann, C., Madsen, T., Hjorthøj, C., Forman, J. L., Kerkhof, A. J., Nordentoft, M., & Erlangsen, A. (2021). Effectiveness of an Internet-based self-help therapy program for suicidal ideation with follow-up at 6 months: results of a randomized controlled trial. *The Journal of Clinical Psychiatry*, *82*(5), 36522. doi: 10.4088/jcp.20m13803

van Spijker, B. A., van Straten, A., & Kerkhof, A. J. (2014). Effectiveness of online self-help for suicidal thoughts: results of a randomised controlled trial. *PloS one*, *9*(2), e90118. https://doi.org/10.1371/journal.pone.0090118

Van Spijker, B. A., Werner-Seidler, A., Batterham, P. J., Mackinnon, A., Calear, A. L., Gosling, J. A., ... & Christensen, H. (2018). Effectiveness of a web-based self-help program for suicidal thinking in an Australian community sample: randomized controlled trial. *Journal of Medical Internet Research*, *20*(2), e15. doi:10.2196/jmir.8595

Wilks, C. R., Lungu, A., Ang, S. Y., Matsumiya, B., Yin, Q., & Linehan, M. M. (2018). A randomized controlled trial of an Internet delivered dialectical behavior therapy skills training for suicidal and heavy episodic drinkers. *Journal of affective disorders*, *232*, 219-228. https://doi.org/10.1016/j.jad.2018.02.053

© 2025 The Author(s). Distributed as a Hogrefe OpenMind article under the license CC BY 4.0 (https://creativecommons.org/licenses/by/4.0)
